# Fingolimod in the treatment algorithm of relapsing remitting multiple sclerosis: a statement of the Central and East European (CEE) MS Expert Group

**DOI:** 10.1007/s10354-012-0123-y

**Published:** 2012-08-16

**Authors:** Franz Fazekas

**Affiliations:** Department of Neurology, Medical University of Graz, Auenbruggerplatz 22, 8036 Graz, Austria

**Keywords:** Multiple sclerosis, Treatment, Immunomodulation, Immunosuppression, Fingolimod, Multiple Sklerose, Behandlung, Immunmodulation, Immunsuppression, Fingolimod

## Abstract

Fingolimod is the first oral treatment of multiple sclerosis. It is the first-in-class sphingosine 1-phosphate receptor modulator that binds to sphingosine 1-phophate receptors on lymphocytes and via downregulation of the receptor prevents lymphocyte egress from lymphoid tissues into the circulation. This mechanism reduces the infiltration of potentially auto-aggressive lymphocytes into the central nervous system. Two large phase III studies with fingolimod have shown superior efficacy of the drug in two dosages compared to placebo and to weekly intramuscular injections of Interferon beta-1a. Among possible side effects of the drug is a transient bradycardia after the first dose of fingolimod including possible AV blockade and therefore monitoring of pulse rate and blood pressure for 6 h following the first application is needed. During treatment, attention has to be given to specific infections, elevated liver enzymes, and ophthalmologic changes. Recommendations on the use of fingolimod including safety aspects are given in this article.

## Abstract (de)

Fingolimod ist das erste oral verabreichbare Medikament zur Behandlung der Multiplen Sklerose. Es ist der erste Sphingosin 1-phosphat Rezeptor Modulator seiner Klasse, der sich an Sphingosin 1-phosphat Rezeptoren auf Lymphozyten bindet und über Abregulation den Austritt von Lymphozyten aus dem lymphatischen Gewebe in die Blutbahn verhindert. Dieser Mechanismus reduziert das Einwandern potentiell autoagressiver Lymphozyten in das Zentralnervensystem. Zwei große Phase-III-Studien mit Fingolimod haben überlegene Wirksamkeit dieser Substanz in zwei Dosierungen gegenüber Plazebo sowie wöchentlicher intramuskulärer Injektion von Interferon beta-1a gezeigt. Zu den möglichen Nebenwirkungen des Medikamentes gehört eine transiente Bradykardie nach der ersten Dosis Fingolimod einschließlich möglicher AV-Blockierung, weshalb über einen Zeitraum von 6 Stunden nach der ersten Verabreichung Pulsrate und Blutdruck monitiert werden sollen. Während der Behandlung müssen spezifische Infektionen, erhöhte Leberenzyme und ophthalmologische Veränderungen Beachtung finden. Dieser Artikel gibt Empfehlungen zum Einsatz von Fingolimod einschließlich der notwendigen Sicherheitsaspekte während der Behandlung mit diesem Medikament.

## Introduction

Multiple sclerosis (MS) is a chronic autoimmune inflammatory disease of the central nervous system (CNS) diagnosed mainly in the third or fourth decade of life. Its course is characterized by inflammation, demyelination, and axonal loss even in the early stages of the disease. Accumulation of these pathological processes is responsible for the clinical disease progression and the patients’ prognosis. MS affects with an average incidence rate of 100 in 100,000 approximately 490,000 individuals in the European Union [1]. Although the exact etiology of MS is still unknown, the current concept derived from animal models assumes that environmental factors may trigger the initiation of an altered immune response in a genetically susceptible individual. The pathophysiology is complex as in other inflammatory and neurodegenerative diseases resulting in unpredictable and variable clinical outcomes.

Most patients initially present with the relapsing–remitting form of MS (RRMS) and may progress to the secondary progressive (SP) form with or without superimposed relapses after variable intervals of time [2]. RRMS patients suffer from episodes of objective neurologic dysfunction for a period of at least 24 h but in most patients these deficits last for weeks to months or remain permanent in some cases [3]. Frequent relapses in the first 2 years of the disease and shorter interattack intervals in this period predict shorter times to reach defined disability endpoints and a shorter time interval to develop SPMS [4]. Disease-modifying drugs (DMDs) can reduce the relapse rate and delay the time of disease progression if treatment is started early [5].

In the last decade, scientific progress in immunology and the discovery of new therapeutic targets for the treatment of MS boosted development programs of new therapeutic agents as well as for drugs already in the market for other indications. These treatment strategies included efforts towards selective immunomodulation/immunosuppression such as that obtained with fingolimod.

This article reflects the outcome of an experts’ meeting involving clinical neurologists experienced in research and treatment of MS from eight European countries to discuss the clinical benefit/risk profile of the first approved oral treatment for MS, fingolimod (Gilenya™), to suggest the position of this new therapy within the current treatment algorithm and to make recommendations for selection and management of patients.

## Established therapies for MS

### Interferon beta and glatiramer acetate (GA)

Over more than 15 years, the first-line treatment of RRMS predominantly consisted of four DMDs: Interferon (IFN) beta-1b (Betaferon^®^ 250 mg s.c. every other day), IFN beta-1a (Avonex^®^ 30 mg i.m. once a week), IFN beta-1a (Rebif^®^ 22 mg or 44 mg s.c. three times a week), and GA (Copaxone^®^ 20 mg s.c. once a day).

These agents have been approved by the European Medicines Agency (EMA) for the treatment of patients with RRMS after successful class I-randomized placebo-controlled trials [6–15]. Further studies in patients who have had a single attack of demyelination (clinical isolated syndrome, CIS), and who were considered to be at high risk for clinically definite MS, led to the approval for Avonex^®^, Betaferon^®^ , and Copaxone^®^ in this early stage of MS [16–20]. Following one positive trial, Betaferon^®^ received approval from the EMA also for the treatment of patients with SPMS and superimposed relapses [21].

The benefit/risk profile of these DMDs was positively assessed on their efficacy to significantly reduce disease activity by reducing the number of relapses and the occurrence of new and enhancing lesions detected by magnetic resonance imaging (MRI) and delaying relapse-related progression of disability compared to placebo. Studies in CIS and data from natural history studies have led to a consensus to consider treatment at least as soon as the diagnosis of MS is established or after a CIS if there is a high risk to develop clinically definite MS.

The moderate efficacy of the DMDs is accompanied by a safety profile with mild to moderate frequent adverse events. For IFN beta products, these comprise of injection site reactions/pain and the post injection flu-like syndrome for 12–24 h which are often transient and subside after the first 6 weeks, and of rare local skin necrosis. With GA injection, site reactions include erythema, pain, and lipoatrophy and the rare post injection systemic reaction, which includes vasodilatation, chest tightness, and shortness of breath lasting 5–15 min, which may occur once during treatment in about 20 % of patients [22–25].

Besides lack of therapeutic efficacy and the inconvenience of side effects, particularly in the first months of treatment, the fact that all first-line DMDs have to be applied by injection limits adherence to long-term treatment with these drugs.

Complete adherence to the dose regimen was found in 75 % of 2,648 patients with average treatment duration of 31 months in a multicenter observational study [26]. Patients and physicians received paper questionnaires regarding adherence to the prescribed treatment regimen. Adherence was defined as not missing a single injection within 4 weeks before the study. The most common reasons for nonadherence were forgetting to administer the injection and other injection-related reasons. Another recently published study assessed the impact of adherence to DMDs on clinical and economic outcomes in a cohort of 2,446 patients [27]. Adherence was assessed in this study using the medication possession ratio (MPR) derived from the administrative claims database. Patients with MPR ³ 80 % were regarded as adherent. A total of 59.6 % of the patients were adherent to their treatment. Adherence was associated with better clinical and economic outcomes including lower risks for MS-related hospitalization, MS relapses, and less MS-related medical costs. In an adherence study comparing data from retrospective self-reports, medication diaries and electronic monitoring of needle disposal, Bruce found that nearly one-fifth of the patients missed more than 20 % of the injections and concludes that studies using self-reports and diaries may underestimate poor adherence [28].

## Mitoxantrone and natalizumab

Two other drugs have been approved in Europe for treatment of MS-patients. Since 2003 mitoxantrone, a synthetic anthracenedione with cytotoxic and immunosuppressive effects is labelled in some European countries for patients with SPMS and an EDSS of 3–6, and for patients with active relapsing progressive MS despite treatment with first-line DMDs. A randomized placebo-controlled trial resulted in a significant effect on relapse rate and disease progression [29]. In a non-randomized subgroup of the study, mitoxantrone did not reduce Gadolinium positive (Gd^+^) MRI scans compared to placebo but resulted in positive trends of secondary MRI outcome measures [30]. Similar to other cytotoxic drugs, mitoxantrone may induce nausea, vomiting, the risk of infections, secondary leukemia [31], amenorrhea, and infertility. Due to its cardiotoxicity, the mitoxantrone use in MS patients is limited to a total cumulative dose of 100–140 mg/m^2^.

Natalizumab was authorized for the treatment of MS patients in June 2006. It is the first monoclonal antibody used in MS. This antibody is directed against the a4 subunit of a4b1 integrin. It is believed that natalizumab acts by blocking the entry of immune cells into the CNS via the interference of the adherence of leukocytes to endothelial vascular cell adhesion molecule (VCAM)-1 [32]. Natalizumab reduced the risk of disability progression over two years by 42 % and the relapse rate by 68 % at one year and 69 % after two years. The accumulation of new or enlarging hyperintense lesions detected by T2-weighted MRI was reduced by 82 % at two years and the number of Gd^+^ MRI lesions by 92 % [33].

A second Phase III study compared the combination of natalizumab with intramuscular INF beta-1a with INF beta-1a monotherapy. The annualized relapse rate was reduced by 54 % with the combination compared to the INF beta-1a only treatment [34]. In this study, the administration of natalizumab had to be suspended during the open-label follow-up study when two cases of progressive multifocal leukoencephalopathy (PML) were identified. PML is a rare and potentially fatal disease of the brain caused by the JC Virus (JCV).

The European Commission granted a marketing authorization in 2006 for second-line treatment of patients with RRMS or for patients with highly active disease. At the time of approval, the risk of developing PML was estimated to be 1/1,000 patients. The last assessment report from EMA in 2010 stated that the risk of developing PML increases after 24 months of treatment and with prior immunosuppressive treatment [35]. The EMA has recently approved the inclusion of anti-JCV antibody status as an additional factor to assess the individual risk of a patient for developing PML before and during treatment with natalizumab. Beside this very rare adverse effect, the safety profile of natalizumab includes some infusion reactions (23.1 % of patients), hypersensitivity reactions (4 % patients), other opportunistic infections, and liver damage. Persistent neutralizing antibodies to natalizumab interfering with the drug’s efficacy were found in 6 % of patients [36].

Approved DMDs are associated with poor adherence, suboptimal therapeutic response and frequent mild to moderate side effects. The use of mitoxantrone in SPMS and progressive relapsing MS is limited due to its dose-related toxicity. Natalizumab, while a potent and effective drug, on clinical and paraclinical parameters of disease activity, is associated with rare cases of the opportunistic CNS infection PML. All these medications have to be given either as a self-injection or as infusion [37]. Therefore much attention has been paid by the MS community to clinical programs with oral treatment including fingolimod, cladribine, fumaric acid, teriflunomide, laquinimod, and others [38–41] which may serve to overcome some of these limitations and to increase adherence.

## Fingolimod

Fingolimod (Gilenya^®^) has been approved by health authorities in the United States and Australia as a first-line treatment for relapsing forms of MS and in Russia, Switzerland, and United Arab Emirates for RRMS.

In January 2011, Fingolimod received a positive opinion from the Committee for Medicinal Products for Human Use (CHMP), a prerequisite for the approval by the European authority. The European Commission granted a marketing authorization valid throughout the European Union for Gilenya^®^ on 17 March 2011. The recommended indication defined by the CHMP is that fingolimod is indicated as single disease-modifying therapy in highly active RRMS for the following adult patient groups:

- Patients with high disease activity despite treatment with a beta-interferon.
  These patients may be defined as those who have failed to respond to a full adequate course (normally at least 1 year of treatment) of beta-interferon. Patients should have had at least one relapse in the previous year while on therapy, and have at least nine T2-hyperintensive lesions on cranial MRI or at least one Gadolinium-enhancing lesion. A “non-responder” could also be defined as a patient with an unchanged or increased relapse rate or ongoing severe relapses, as compared to the previous year.
  or
- Patients with rapidly evolving severe RRMS defined by 2 or more disabling relapses in 1 year, and with one or more Gadolinium enhancing lesions on brain MRI or a significant increase in T2 lesion load as compared to a recent MRI.

### Mode of action

Fingolimod is the first orally bioavailable sphingosine 1-phosphate (S1P) receptor modulator. S1P is derived from sphingosine, phosphorylated by ubiquitously appearing sphingosine-kinases. S1P binds to five cell-surface high-affinity G-protein-coupled receptors, the S1P_1–5_. Distribution and signalling function varies between the subtypes. S1P_1–3_ are mainly distributed in the immune system, CNS and cardiovascular organs, S1P_4_ in lymphoid tissue and S1P_5_ in CNS white matter [42, 43].

S1P and its receptors regulate circulation of lymphocytes between blood and lymphoid organs depending on specific requirements of the immune system and accomplished by enzymes that regulate sphingolipid metabolism and partly by a concentration gradient between lymphoid organs and blood [43].

**Fig. 1:**
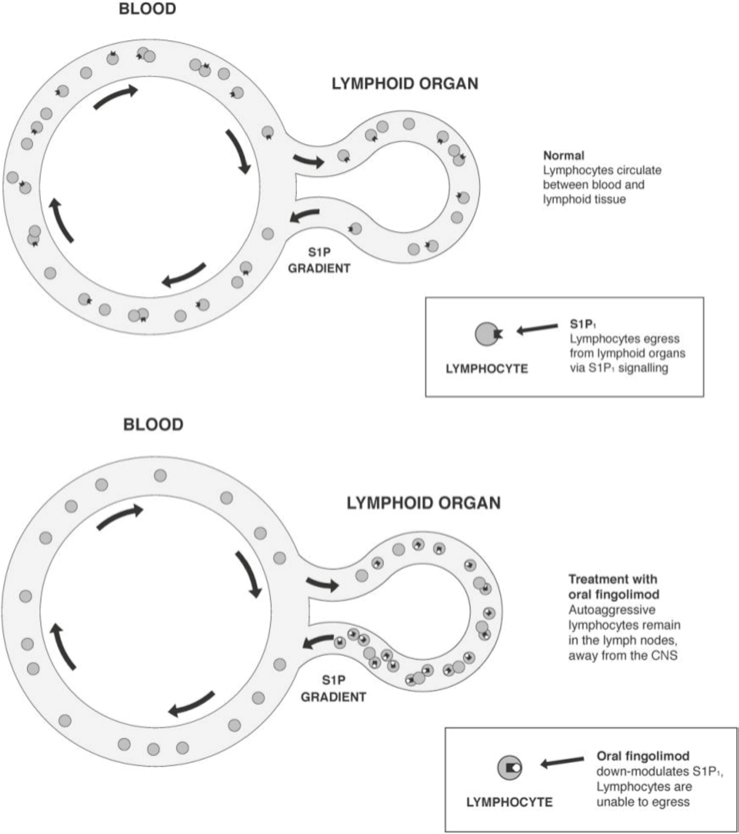
Mode of action of Fingolimod down-regulates S1P_1_. Lymphocytes remain in lymphoid tissue. (Modified from [42])

Specifically S1P_1_ on lymphocytes regulates homing and egress of lymphocytes in and from lymphoid organs. The active phosphorylated form of fingolimod binds with high affinity to S1P_1_ and to a less extent to S1P_3–5_. For the treatment of MS, the most important consequence of the down-regulation (internalization) of S1P_1_ is that T-lymphocytes, including potentially auto-aggressive T cells, remain retained in lymph nodes and their number in the circulation is considerably reduced (Fig. 1).

Experimental and clinical data indicate that fingolimod retains naïve T cells and central memory T cells (TCMs) including Th17 cells in lymphoid tissue. The proinflammatory Th17 cells may have a central role in CNS inflammation [44]. In a small prospective observational study, fingolimod reduced the number of Th17 (IL17 producing cells) by > 90 % [45]. The retention of lymphocytes does not lead to an enlargement of lymph nodes since normally only 2 % of the total number of these cells circulate in the blood.

Fingolimod crosses the blood–brain barrier and the oral formulation can result in biologically active concentration in the CNS. It is likely that the drug interacts directly with S1P receptors on neurons, oligodendrocytes, astrocytes, and their progenitor cells. In rodent experimental autoimmune encephalomyelitis models, fingolimod has demonstrated prophylactic and therapeutic efficacy, reversing central inflammation, favouring preservation of the integrity of the blood–brain barrier, and inducing structural and functional restoration of the CNS parenchyma [42, 46–50]. Ongoing preclinical and clinical studies are looking at whether the direct interaction with S1P receptors in the CNS contributes to the clinical efficacy of the drug and can provide a clinically relevant reduction of neurodegenerative processes or initiate repair mechanisms in MS patients [50].

### Efficacy in clinical trials

Clinical efficacy and safety of Fingolimod have been evaluated in an extensive development program which has been already reviewed elsewhere [50–52]. In short, the phase II study program consisted of a six-months placebo-controlled core study of 281 patients with relapsing MS and a six-months extension switching placebo patients to active treatment which showed a significant reduction in the detection of new MS lesions on MRI and of clinical disease activity for both daily doses of 1.25 or 5.0 mg of fingolimod [53]. In the subsequent follow-up of these patients, all patients receiving fingolimod 5.0 mg were switched to 1.25 mg during months 15–24 with no indication that lowering the dose from 5.0 to 1.25 mg was associated with a reduction of efficacy [54]. This consistent therapeutic effect was also confirmed after 36 months [55].

The phase III program consisted of two large trials, the placebo-controlled FREEDOMS (FTY Research Evaluating Effects of Daily Oral therapy in Multiple Sclerosis) and the TRANSFORMS (Trial Assessing Injectable Interferon versus FTY720 Oral in Relapsing–Remitting Multiple Sclerosis) studies.

In FREEDOMS, which included a total of 1,272 RRMS patients, all clinical and MRI efficacy endpoints significantly favoured both active-treated groups over placebo with no difference in efficacy between the two fingolimod doses after 24 months [56]. The annualized relapse rate (ARR) was 0.18 with 0.5 mg of fingolimod, 0.16 with 1.25 mg fingolimod, and 0.4 with placebo (*p* < 0.001 for both fingolimod doses versus placebo). Fingolimod reduced the risk of disability progression, confirmed after 3 months, over the 24-months period (hazard ratios were 0.70 for the 0.5 mg dose and 0.68 for the 1.25 mg dose; *p* = 0.02 vs placebo, for both comparisons). The cumulative probability of disability progression (confirmed after 3 months) was 17.7 % for fingolimod 0.5 mg, 16.6 % for fingolimod 1.25 mg, and 24.1 % for placebo. The risk of disability progression confirmed after 6 months was also reduced with both doses of fingolimod over the study period. EDSS scores and MSFC z-scores remained stable or improved slightly in the active treatment groups and worsened in the placebo group. Both fingolimod doses were also superior to placebo with regard to MRI-related measures. Actively treated patients had significantly fewer Gd^+^ lesions than patients on placebo (mean 0.2 vs 1.1) and significantly fewer new or enlarged lesions on T2-weighted MRI scans at 24 months (mean 2.5 vs 9.8). Ninety percent of actively treated patients were free of Gd^+^ lesions compared to 65 % of placebo-treated patients. Beneficial effects of fingolimod were also noted on the volume changes in lesions on T2- and T1-weighted scans and brain volume reduction was significantly smaller with fingolimod [56].

**Fig. 2:**
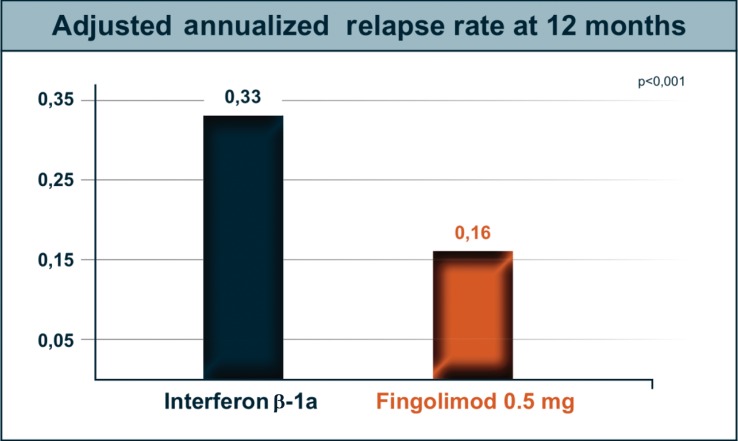
Adjusted annualized relapse rate in the TRANSFORMS study comparing the efficacy of Fingolimod with interferon-beta 1a i.m. (Modified from [57])

TRANSFORMS compared the efficacy of fingolimod 0.5 or 1.25 mg daily with that of IFN beta-1a at weekly doses of 30 mg IM over a 12-months period in 1,292 patients with RRMS [57]. The ARR defined as the primary efficacy endpoint was significantly lower in both groups receiving fingolimod compared with the INF beta-1a group (0.20 in the 1.25 mg group, 0.16 in the 0.5 mg group—Fig. 2—and 0.33 in the INF beta-1a group; *p* < 0.001). Significantly more relapse-free patients were found in the two fingolimod groups compared to INF beta-1a-treated patients (79.8 % for 1.25 mg, and 82.6 % for 0.5 mg vs 69.3% for INF beta-1a; *p* < 0.001). Confirmed disability progression was infrequent in all study groups. There were no significant differences in the time to progression of disability or in the proportion of patients with confirmed progression among the study groups. MRI findings supported the primary clinical results. Patients in the two fingolimod groups had significantly fewer new or enlarged hyperintense lesions on T2-weighted images (1.5 for 1.25 mg, 1.7 for 0.5 mg, and 2.6 for INF beta-1a) at 12 months compared to the INF beta-1a group and fewer Gd^+^ lesions (0.23, 0.14 vs 0.51). The mean percent reduction in brain volume from baseline to 12 months was significantly lower in the two fingolimod groups than in the INF beta-1a group [57].

In the TRANSFORMS extension study switching from INF beta-1a to fingolimod led to enhanced efficacy while continuous treatment with fingolimod over 2 years provided a sustained treatment effect [58].

### Safety in clinical trials

The most frequent reported adverse events in MS-studies with fingolimod 0.5 mg were influenza viral infections, nasopharyngitis, fatigue, back pain, diarrhea, bronchitis, and nausea. The most common laboratory abnormalities observed with fingolimod were lymphopenia and abnormal liver function tests. In the FREEDOMS study, treatment was discontinued due to adverse events in 7.5 % of patients on fingolimod 0.5 mg, in 14.2 % of patients on fingolimod 1.25 mg, and in 7.7 % of patients on placebo, the rate of any serious adverse event was 10.1, 11.9, and 13.4 %, respectively [56].

The overall rate of infections was similar in the fingolimod and placebo groups. Lower respiratory tract infections were more frequent in the fingolimod groups compared to the placebo group (9.6, 11.4 vs 6.0 %). After the first month of the trial, the blood lymphocyte counts were reduced by 73 % with fingolimod 0.5 mg and by 76 % with fingolimod 1.25 mg.

After the first dose of fingolimod, heart rate decreased with a maximum reduction of resting pulse rate of 8 bpm with fingolimod 0.5 mg and 10 bpm with fingolimod 1.25 mg. Bradycardia was reported in nine patients on fingolimod 0.5 mg and in 14 on fingolimod 1.25 mg. Seven of these cases were assessed as serious adverse events and resolved within 24 h without treatment. Two patients from the fingolimod 0.5 mg group, six patients from the fingolimod 1.25 mg group, and three patients of the placebo group developed first- or second-degree atrioventricular (AV) block. During extended treatment, no effects on heart rate were observed.

Macular edema was reported in 7 patients of the fingolimod 1.25 mg group. The majority of the cases were diagnosed within the first 3 months of the study and resolved within 6 months after discontinuation of fingolimod.

Malignant neoplasms were reported in four patients on fingolimod 0.5 mg, four receiving fingolimod 1.25 mg, and in ten patients of the placebo group. Eleven of these cases were skin cancers (basal-cell carcinoma, malignant melanoma, or Bowen’s disease), three cases in the fingolimod 1.25 mg group, four cases in the 0.5 mg group, and four with placebo. All were removed successfully.

**Fig. 3:**
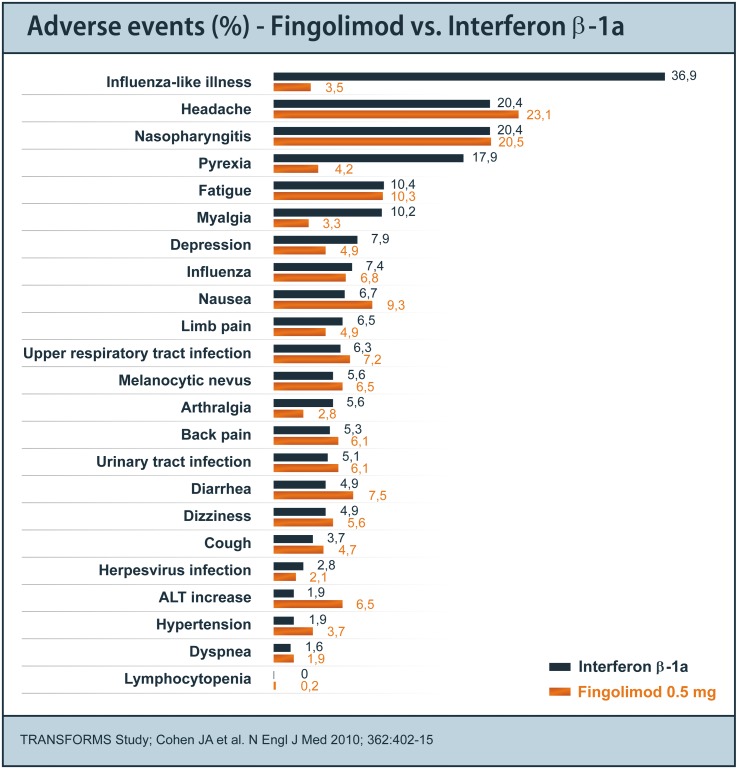
Adverse event profile in the TRANSFORMS study comparing the efficacy of Fingolimod with interferon-beta 1a i.m. (Modified from [57])

The safety profile of the TRANSFORMS trial (Fig. 3) was very similar to the FREEDOMS study with the addition of two cases of macular edema and one case of second-degree AV Block in the 0.5 mg fingolimod group [57].

## Treatment considerations and recommendations

### Special safety areas

Based on the pharmacodynamic properties of fingolimod and its mode of action in MS, special safety areas have to be mentioned and closely monitored:

- Heart rate and AV conduction at treatment initiation
- Infections
- Risk of macular edema
- Liver transaminase elevation
- Reproductive toxicity
- Concomitant or prior use of immunosuppressive drugs.

### Cardiac safety (see addendum)

Initiation of treatment with fingolimod results in a transient decrease in heart rate and may induce AV conduction delays (AV block first or second degree). This also applies to recommencing treatment after an interruption of more than 14 days. After the first dose, the decline in heart rate starts within 1 h and is maximal at approximately 4–5 h. With continued administration, heart rate returns to baseline within 1 month. Conduction abnormalities were usually asymptomatic, did not require treatment, and resolved within 24 h (in the study, two patients were treated with atropine and one patient was treated with isoproterenol).

#### Recommendations (see addendum)

In patients with bradycardia (< 55 bpm), AV conduction delays, sick sinus syndrome, ischemic heart disease or congestive heart failure, advice from a cardiologist is recommended before initiating treatment. Treatment with fingolimod should not be initiated while patients take Class Ia (e.g. quinidine, procainamide) or Class III (e.g. amiodarone, sotalol) antiarrhythmic drugs.

Patients receiving beta-blockers or other substances which may reduce heart rate may have an increased risk of bradycardia because of additive effects of fingolimod on heart rate.

All patients should be observed clinically for a period of at least 6 h for signs and symptoms of bradycardia following the initial administration of the drug. Should post-dose bradycardia-related symptoms occur, appropriate clinical management should be initiated and observation should continue until symptoms have resolved and the heart rate is in the normal range.

### Infections

Fingolimod causes a dose-dependent reduction in peripheral lymphocyte count to 20–30 % of baseline levels because of the reversible sequestration of lymphocytes in lymphoid tissues. Fingolimod may therefore increase the risk of infections, some serious in nature. In MS studies, the overall rate of infections (72 %) and serious infections (2 %) with the 0.5 mg dose was similar to placebo. However, lower respiratory tract infections, primarily bronchitis and, to a lesser extent, pneumonia were more common in fingolimod-treated patients.

Two fatal cases of herpes infections occurred with the 1.25 mg dose: a case of herpes simplex encephalitis in a patient in whom initiation of acyclovir treatment was delayed by one week, and a case of primary disseminated varicella zoster infection in a patient not previously exposed to varicella receiving concomitant high-dose steroid therapy for an MS relapse. Even though fatal infection occurred only in the high-dose group, opportunistic infection could also happen with lower doses.

#### Recommendations

A complete blood count should be obtained before initiation of treatment, at month 1, 3, and 6, and periodically thereafter to check for abnormalities. An absolute lymphocyte count of < 0.2 ´ 10^9^/L should lead to treatment interruption until recovery.

Initiation of treatment with fingolimod should be delayed in patients with severe active infection until recovery.

Patients should be instructed to report symptoms of infections during treatment and till 2 months after treatment discontinuation. Diagnostic measures and treatment for infections should be started in due time if indicated. Suspending fingolimod treatment should be considered during serious infections and consideration of benefit–risk should be undertaken prior to reinitiation of therapy.

Patients without a history of chickenpox or without vaccination against varicella zoster virus (VZV) should be tested for VZV antibodies. If negative, VZV vaccination should be considered and treatment with fingolimod should be postponed until full effect of vaccination has been achieved.

### Macular edema

Macular edema with or without visual symptoms has been reported in 0.4 % of patients treated with fingolimod 0.5 mg and in 1.1 % of patients with the higher dose of 1.25 mg, predominantly in the first 3 to 4 months of treatment. Some patients presented with blurred vision or decreased visual acuity, but others were asymptomatic and diagnosed at routine ophthalmological examination. The macular edema generally improved or resolved spontaneously after discontinuation of fingolimod treatment.

#### Recommendations

Patients with a history of uveitis and patients with diabetes mellitus are at increased risk of macular edema. It is recommended that MS patients with a history of uveitis or diabetes mellitus undergo an ophthalmologic evaluation before initiating fingolimod treatment and have follow-up investigations during treatment.

Other patients should have an ophthalmologic evaluation 3 to 4 months after the initiation of treatment and at any time symptoms may occur during treatment.

It is recommended that treatment with fingolimod should be discontinued if a patient develops macular edema. Whether treatment should be reinitiated after resolution of macular edema depends on the risk–benefit evaluation of the individual patient.

### Hepatic function

During clinical trials, fingolimod 0.5 mg was associated with a threefold or greater elevation in liver transaminases in 8 % of treated patients compared to 2 % of the placebo patients. The mechanism of this effect has not been identified. The elevation of liver enzymes was generally asymptomatic, observed after 3 to 4 months of treatment and turned to normal within approximately 2 months after discontinuation of fingolimod treatment.

#### Recommendations

Recent (< 6 months) transaminases and bilirubin levels should be available before initiation of treatment with fingolimod. Liver transaminases should be monitored at month 1, 3 and 6, and periodically thereafter. With repeated confirmation of liver transaminases above five times the upper limit of normal, treatment with fingolimod should be interrupted.

In patients who develop symptoms suggestive of hepatic dysfunction such as unexplained nausea, vomiting, abdominal pain, fatigue or jaundice, the liver enzymes should be checked and fingolimod should be discontinued if significant liver injury is confirmed.

Patients with severe preexisting hepatic impairment should not be treated with fingolimod.

### Reproductive toxicity

Animal studies have shown reproductive toxicity including fetal loss and organ defects. S1P receptors are known to be involved in vascular formation during embryogenesis.

#### Recommendations

Women of childbearing potential should be advised on the potential serious risk for the fetus and the need of effective contraception during treatment with fingolimod. Since elimination of fingolimod takes about 2 months after the end of treatment, the potential risk for the fetus may persist and contraception should be continued over this time.

Before initiation of treatment in women with childbearing potential, a negative pregnancy test result is necessary.

If a woman becomes pregnant while on treatment with fingolimod, discontinuation of treatment is recommended.

### Prior immunotherapy

Clinical trial data suggest that no wash-out period is needed when switching from INF beta or GA to fingolimod if any immune effects of such therapies have resolved. In the clinical trials, patients were excluded if treated with natalizumab, other monoclonal antibodies or cytotoxic drugs in less than 6 months prior to fingolimod therapy [56, 57].

#### Recommendations

Natalizumab treatment should be stopped for at least 2 to 3 months before treatment initiation with fingolimod so as to avoid the risk of cumulative immunosuppression from the 70 % decrease in total lymphocyte count with fingolimod. Cytotoxic drugs (e.g. mitoxantrone) should be washed out for at least 6 months before commencement of treatment with fingolimod.

A detailed patient management plan for treatment of RRMS with fingolimod is provided in Table 1.

**Table 1:**
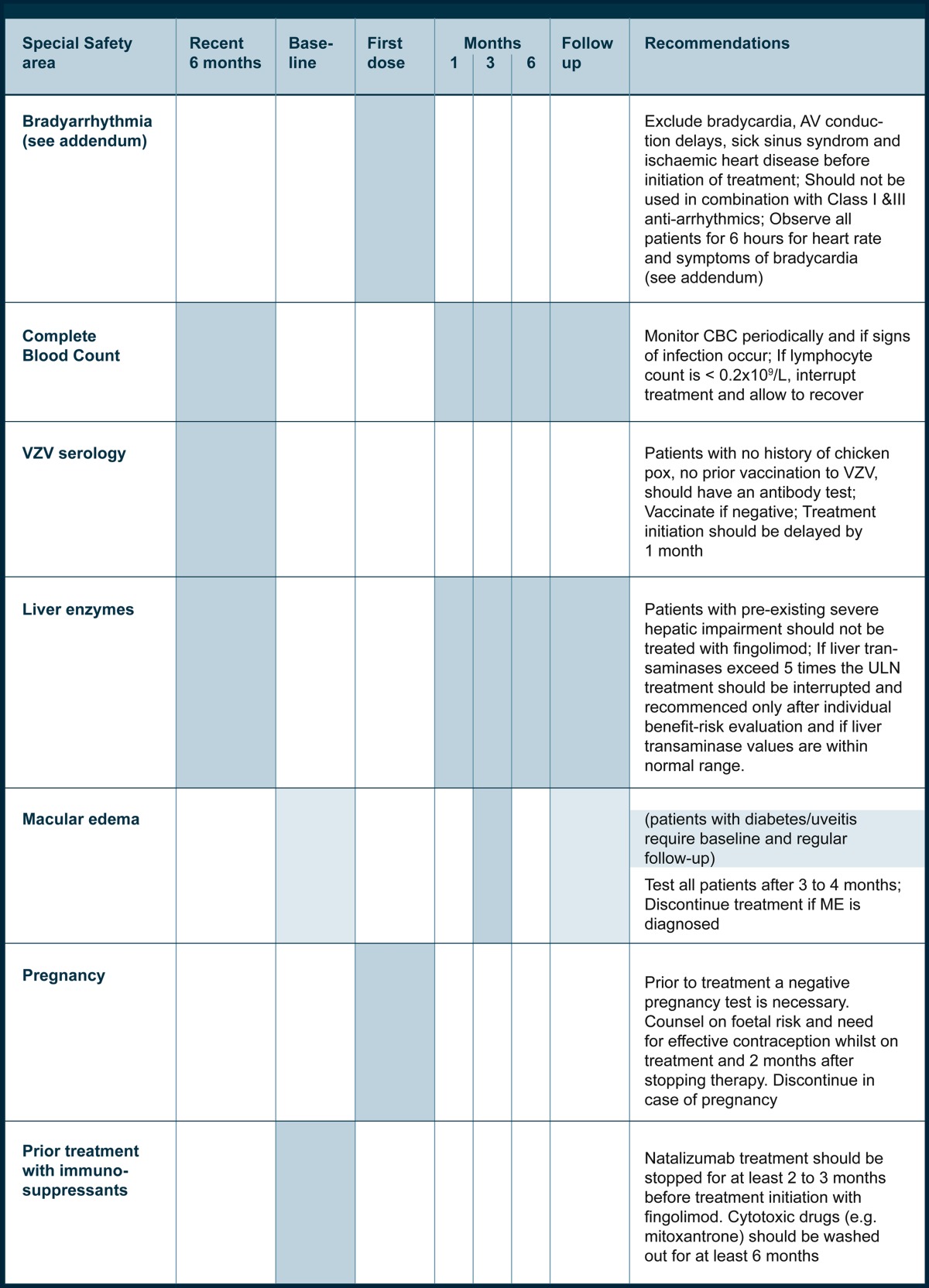

### Selection and management of patients

Fingolimod 0.5 mg daily is the first oral drug for the treatment of RRMS. At present, it is approved as a first-line treatment for relapsing forms of MS in the United States, in Russia, Switzerland, and in the United Arab Emirates.

In Europe, the CHMP defined the indication for fingolimod as a disease-modifying monotherapy in highly active RRMS:

- For patients with high disease activity despite treatment with an INF beta (non-responders, treatment failure) and
- For patients with rapidly evolving severe RRMS without prior treatment.

**Fig. 4:**
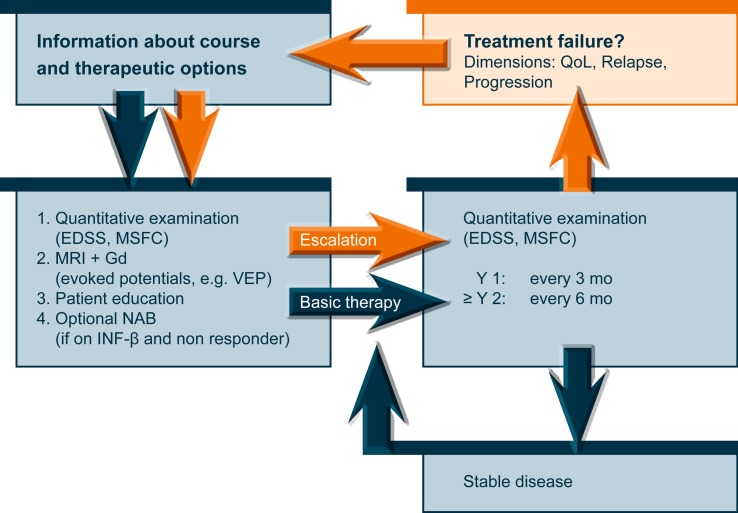
Patient evaluation scheme in the concept of escalating immunotherapy of RRMS. (Modified from [59])

**Fig. 5:**
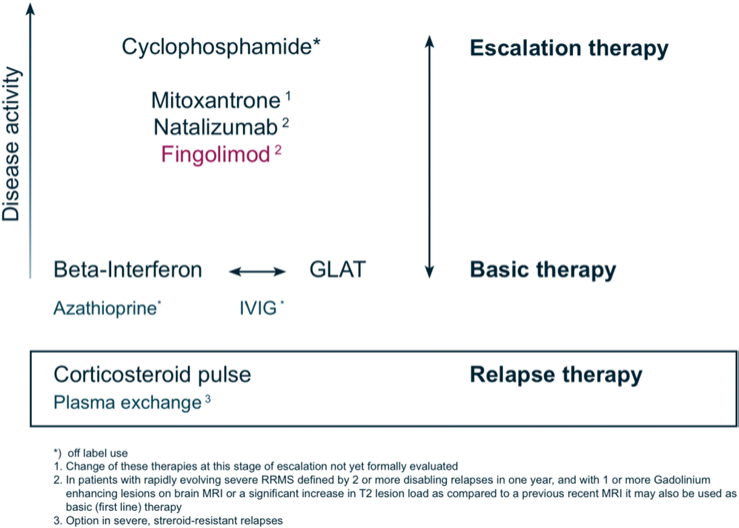
Current options of escalating immunotherapy for RRMS. (Modified from [59])

From a clinical point of view, treatment failure is defined as continuing disease activity (in the form of relapses supported by new or active MRI lesions) and progression of disability. According to expert opinion this applies in a similar manner to prior treatment with GA as to that with INF beta. In clinical practice, unbearable side effects and low compliance also constitute a kind of treatment failure. Since no combinations of DMDs and immunosuppressant or cytotoxic drugs are approved in the case of a treatment failure and no robust clinical studies on the efficacy of such combinations are published, the concept of escalating immunotherapy of the Multiple Sclerosis Therapy Consensus Group is still appropriate for this situation [59]. The proposed algorithm for patient evaluation and decision-making is provided in Fig. 4. In a hierarchy of the existing approved and labelled treatments of RRMS, fingolimod is positioned equal to natalizumab in Europe (Fig. 5). The reason for the identically labelled indications of the two substances in Europe may on one hand come from their comparable efficacy in clinical and MRI endpoints of clinical studies. On the other hand, the potential risk of fingolimod treatment may have been assessed with caution while long-term experience, as with any new drug before introduction into clinical practice, is limited. Excluding the patients at risk by careful pretreatment examination and follow-up according to the above recommendations will contribute to the safety of fingolimod in clinical practice and serve to maximize patients’ benefits from the advantages of this new drug.

In conclusion, large phase III studies with fingolimod have shown favorable efficacy compared to placebo and to a standard treatment with INF beta-1a i.m. and an acceptable safety profile. The position of fingolimod in clinical practice will be influenced by issues of long-term adherence, quality of life, and long-term safety of patients. To ensure best use of this new treatment for patients with RRMS, treatment should be initiated by experienced MS-centers and monitored and documented according to the recommendations in the six special safety areas.

#### Acknowledgements

The authors thank Siegfried Mayerhofer from Cytro medical and clinical project management GmbH for medical writing and editorial assistance for the manuscript. This assistance and the meeting of the expert group were funded by Novartis Pharma GmbH.

## Addendum

Following submission of this manuscript the EMA started a review of the cardiovascular safety of Gilenya following receipt of information related to an unexplained sudden death in a patient within 24 h of taking Gilenya for the first time in January 2012 [60]. The Agency reviewed all available data on the heart safety of Gilenya, including 15 cases of sudden or unexplained death in patients treated with Gilenya. It noted that most of the deaths and cardiovascular problems had occurred in patients with a history of cardiovascular problems or taking other medicines. However, the data reviewed were not conclusive as to whether Gilenya was the cause of the deaths. Therefore, the EMA was of the opinion that the possible risk of heart problems in patients taking Gilenya could be minimized by further strengthening the existing warnings on the cardiovascular effects of the medicine and ensuring close monitoring of all patients as follows:

Treatment with Gilenya is not recommended [61]**:**

- In patients with a history of cardiovascular or cerebrovascular disease. However, if treatment with Gilenya is considered necessary, advice from a cardiologist should be sought regarding the appropriate heart monitoring for these patients when starting treatment. Monitoring should be at least overnight;
- In patients taking certain antiarrhythmic medicines (medicines used to restore normal cardiac rhythm);
- In patients taking certain medicines that lower the heart rate. However, if treatment with Gilenya is considered necessary, advice from a cardiologist should be sought as to whether these patients should be switched to a different medicine that does not lower the heart rate, or whether they should be continuously monitored overnight by ECG after the first dose.

When starting treatment with Gilenya, doctors should:

- Before the first dose, check the patient’s blood pressure, heart rate, as well as their heart by ECG;
- After the first dose, check the patient’s blood pressure and heart rate every hour for 6 h;
- Doctors are recommended to continuously monitor the patient’s heart function by ECG for 6 h after the first dose.

Doctors are recommended to extend monitoring after the 6-h period if:

- At the end of the 6-h period, the heart rate is at its lowest since taking the first dose. In this case, the monitoring should be extended for at least two more hours and until the heart rate increases again;
- Patients develop any clinically relevant heart problem (such as bradycardia or AV block). If so, doctors are advised to extend the monitoring period at least overnight and until resolution.

With these risk-minimization measures in place, the Agency concluded that the benefits of Gilenya continue to outweigh the risk and updated the Gilenya® prescribing information [60].

### Conflict of interest

Thomas Berger has participated in meetings sponsored by and received honoraria (lectures, advisory boards, consultations) from pharmaceutical companies marketing treatments for MS: Allergan, AOP, Baxter, Bayer (Schering), Biogen-Idec, Biotest, CSL Behring, Merck (Serono), Novartis, Sanofi Aventis, TEVA. His institution has received financial support by unrestricted research grants (Allergan, AOP, Biogen-Idec, Berlex, Bayer, Biotest, CSL Behring, Merck Serono, Sanofi Aventis) and for participation in clinical trials in MS sponsored by Bayer Schering, Biogen-Idec, Merck Serono, Novartis, Octapharma, Roche, Sanofi Aventis, Teva.

Franz Fazekas serves on scientific advisory boards for Bayer Schering, Biogen Idec, Genzyme, Merck Serono, Novartis, and Teva Pharmaceutical Industries Ltd./Sanofi Aventis and has received speaker honoraria from Biogen Idec, Merck Serono, Novartis, and Sanofi-Aventis.

Eva Havrdová has received speaker honoraria and payments for consulting services and clinical trials from Biogen Idec, Bayer, Genzyme, GSK, Merck Serono, Novartis, and Teva.

Tanja Hojs Fabjan declares no conflicts of interest.

Alenka Horvat Ledinek declares no conflicts of interest.

Gábor Jakab declares no conflicts of interest.

Samuel Komoly has received honoraria for talks and payment for occasional consultancy or research funding from TEVA, Bayer—Schering, Serono, Biogen which manufacture immunomodulatory drugs used in MS.

Jörg Kraus received financial support for research activities from Biogen Idec, Bayer, Genzyme, Sanofi-Aventis, Merck Serono and Novartis. JK received personal compensation from Biogen Idec, Bayer, Sanofi-Aventis, Merck Serono and Novartis for lectures, advisory board participations and consultations.

Egon Kurča declares no conflicts of interest.

Theodoros Kyriakides declares no conflicts of interest.

Ĺubomír Lisý declares no conflicts of interest.

Ivan Milanov declares no conflicts of interest.

Panayiotou Panayiotis declares no conflicts of interest.

Sasa Sega Jazbec declares no conflicts of interest.

Radomír Taláb declares no conflicts of interest.

Latchezar Traykov has received (500–1000 EUR or up to 1500 EUR) honoraria in advisory board fees and lecturer fees from Novartis, Pfizer, GSK, UCB, Gedeon Richter, Actavis, CSC Pharmaceuticals.

Turčáni Peter declares no conflicts of interest.

Karl Vass received honoraria for lectures and participations at advisory boards from Allergan, BayerSchering, Biogen Idec, MerckSerono, Novartis, SanofiAventis and Genzyme.

Norbert Vella has been the recipient of honoraria from Novartis Pharma, financial support to attend meetings from Bial, Biogen Idec, GSK, Merz and Novartis.
